# Supplementary material for: Validation and description of two new north-western Australian Rainbow skinks with multispecies coalescent methods and morphology
Source: PeerJ. 2017 Aug 29;5:e3724. doi: 10.7717/peerj.3724 (PMC5580384; doi:10.7717/peerj.3724)
Supplement: Table S1 — Tissue and specimens list with museum origin, mtDNA lineage, for which analysis samples were used (SD, species delimitation, M, morphology), sex information if available and location. ∗ Correspond to the genetically discordant sample with mtDNA of Triacantha B but nuclear of Triacantha A (with no evidence of admixture in Afonso Silva et al. (2017)). [file peerj-05-3724-s001.docx]

Supplemental Table S1 – Tissue and specimens list with museum origin, mtDNA lineage, for which analysis samples were used (SD – species delimitation, M – morphology), sex information if available and location. * Correspond to the genetically discordant sample with mtDNA of Triacantha B but nuclear of Triacantha A (with no evidence of admixture in Afonso Silva *et al.*, 2017).

| **Tissue/Specimen number** | **Specimen location** | **mtDNA lineage** | **Analysis** | **Sex** | **Latitude** | **Longitude** | **Location** |
| --- | --- | --- | --- | --- | --- | --- | --- |
| BP00664 | Western Australian Museum | Johnstonei A | SD | NA | -14.97630 | 124.91310 | Coronation Island |
| BP01423 | Western Australian Museum | Johnstonei A | SD | NA | -15.94970 | 124.56110 | Storr Island |
| CCM0737 | Australian National University | Johnstonei A | M | NA | -15.19890 | 125.90350 | Spring Creek |
| CCM0762 | Australian National University | Johnstonei A | M | NA | -15.35220 | 126.58810 | Carson escarpment |
| CCM0763 | Australian National University | Johnstonei A | M | NA | -15.33810 | 126.58910 | Carson escarpment |
| CCM0933 | Australian National University | Johnstonei A | M | Female | -14.51903 | 126.45811 | Theda Station |
| CCM0999 | Australian National University | Johnstonei A | M | Female | -14.88556 | 126.35986 | Theda Station |
| CCM1125 | Australian National University | Johnstonei A | SD/M | Female | -14.78060 | 126.63690 | Langoor Hill |
| CCM1126 | Australian National University | Johnstonei A | M | Female | -14.78060 | 126.63690 | Theda Station |
| CCM1127 | Australian National University | Johnstonei A | M | Female | -14.78680 | 126.63420 | Theda Station |
| CCM1128 | Australian National University | Johnstonei A | M | Female | -14.78680 | 126.63420 | Theda Station |
| CCM1132 | Australian National University | Johnstonei A | M | Male | -14.78290 | 126.63510 | Theda Station |
| CCM1133 | Australian National University | Johnstonei A | M | Male | -14.78290 | 126.63510 | Langoor Hill |
| CCM1195 | Australian National University | Johnstonei A | M | Female | -14.82110 | 125.72140 | Mitchell Plateau |
| CCM1196 | Australian National University | Johnstonei A | M | Female | -14.82110 | 125.72140 | Mitchell Plateau |
| CCM1197 | Australian National University | Johnstonei A | M | NA | -14.82110 | 125.72110 | Mitchell Plateau |
| CCM1200 | Australian National University | Johnstonei A | M | Female | -14.83170 | 125.71920 | Mitchell Plateau |
| CCM1264 | Australian National University | Johnstonei A | M | Female | -17.06837 | 125.24618 | Silent Grove |
| CCM1290 | Australian National University | Johnstonei A | M | Female | -16.78641 | 124.92006 | Mt Matthew Gorge |
| CCM1301 | Australian National University | Johnstonei A | M | Female | -16.97111 | 125.02998 | Mt Hart |
| CCM1499 | Australian National University | Johnstonei A | M | NA | -16.99694 | 125.20469 | Bell Gorge Creek |
| CCM1761 | Australian National University | Johnstonei A | M | Female | -14.79721 | 126.50038 | Theda Station |
| CCM1762 | Australian National University | Johnstonei A | M | Female | -14.79721 | 126.50038 | Theda Station |
| D76985 | Museum Vitoria | Johnstonei A | M | NA | -16.99920 | 125.20680 | Bell Gorge |
| D76986 | Museum Vitoria | Johnstonei A | M | NA | -16.99920 | 125.20680 | Bell Gorge |
| R106285 | Western Australian Museum | Johnstonei A | M | NA | -15.43333 | 124.60000 | Augustus Island |
| R113992 | Western Australian Museum | Johnstonei A | M | NA | -15.35000 | 126.61667 | Carson Escarpment |
| R117719 | Western Australian Museum | Johnstonei A | M | Male | -14.16389 | 125.67972 | Descartes Island |
| R117726 | Western Australian Museum | Johnstonei A | M | NA | -14.16380 | 125.67970 | Descartes Island |
| R117760 | Western Australian Museum | Johnstonei A | M | NA | -14.16389 | 125.67972 | Descartes Island |
| R117772 | Western Australian Museum | Johnstonei A | M | NA | -14.38020 | 125.95110 | South West Osborn Island |
| R117787 | Western Australian Museum | Johnstonei A | M | NA | -14.38028 | 125.95111 | South West Osborn Island |
| R117793 | Western Australian Museum | Johnstonei A | M | Male | -14.37972 | 125.94917 | South West Osborn Island |
| R117799 | Western Australian Museum | Johnstonei A | M | NA | -14.34333 | 126.02083 | Middle Osborn Island |
| R117801 | Western Australian Museum | Johnstonei A | M | NA | -14.34194 | 126.02111 | Middle Osborn Island |
| R117811 | Western Australian Museum | Johnstonei A | M | NA | -14.37556 | 125.98972 | Carlia Island |
| R117812 | Western Australian Museum | Johnstonei A | M | NA | -14.33833 | 125.97694 | Kidney Island |
| R117813 | Western Australian Museum | Johnstonei A | M | NA | -14.33833 | 125.97694 | Kidney Island |
| R117822 | Western Australian Museum | Johnstonei A | M | NA | -14.37667 | 125.98972 | Carlia Island |
| R117838 | Western Australian Museum | Johnstonei A | M | NA | -14.37550 | 125.98970 | Carlia Island |
| R117843 | Western Australian Museum | Johnstonei A | M | NA | -14.34917 | 125.95917 | South West Osborn Island |
| R117844 | Western Australian Museum | Johnstonei A | M | NA | -14.34910 | 125.95920 | South West Osborn Island |
| R117851 | Western Australian Museum | Johnstonei A | M | Male | -14.34917 | 125.95917 | South West Osborn Island |
| R117855 | Western Australian Museum | Johnstonei A | M | NA | -14.44694 | 125.99722 | Steep Head Island |
| R158783 | Western Australian Museum | Johnstonei A | M | Female | -14.60194 | 125.20389 | Bigge Island |
| R158784 | Western Australian Museum | Johnstonei A | M | Female | -14.60194 | 125.20389 | Bigge Island |
| R158915 | Western Australian Museum | Johnstonei A | M | Female | -14.61833 | 125.24111 | Purrungku Island |
| R167192 | Western Australian Museum | Johnstonei A | M | Female | -15.37139 | 124.94333 | Coronation Island |
| R167235 | Western Australian Museum | Johnstonei A | M | NA | -15.04889 | 124.95000 | Gray Island |
| R167242 | Western Australian Museum | Johnstonei A | M | Male | -15.06278 | 124.96194 | Glauert Island |
| R167260 | Western Australian Museum | Johnstonei A | M | Female | -15.11639 | 124.92417 | Prince Regent River |
| R167423 | Western Australian Museum | Johnstonei A | M | Female | -15.09222 | 125.12139 | Boongaree Island |
| R167431 | Western Australian Museum | Johnstonei A | M | Male | -15.08806 | 125.17083 | Boongaree Island |
| R167860 | Western Australian Museum | Johnstonei A | M | NA | -14.82389 | 125.70889 | Mitchell Plateau |
| R168219 | Western Australian Museum | Johnstonei A | M | Male | -14.60000 | 125.11667 | Bigge Island |
| R168651 | Western Australian Museum | Johnstonei A | M | NA | -14.46667 | 125.53333 | Katers Island |
| R168803 | Western Australian Museum | Johnstonei A | M | NA | -14.31667 | 126.00000 | Middle Osborn Island |
| R168923 | Western Australian Museum | Johnstonei A | M | Male | -15.08333 | 125.20000 | Boongaree Island |
| R171227 | Western Australian Museum | Johnstonei A | M | Male | -15.35139 | 124.52694 | Augustus Island |
| R171230 | Western Australian Museum | Johnstonei A | M | NA | -15.29056 | 124.39917 | Darcy Island |
| R171237 | Western Australian Museum | Johnstonei A | SD/M | NA | -15.25972 | 124.44528 | Darcy Island |
| R171238 | Western Australian Museum | Johnstonei A | M | Male | -15.38556 | 124.36194 | Byam Martin Island |
| R171242 | Western Australian Museum | Johnstonei A | M | Female | -15.38556 | 124.36194 | Byam Martin Island |
| R171243 | Western Australian Museum | Johnstonei A | M | NA | -15.35806 | 124.99611 | Saint Andrew Island |
| R171488 | Western Australian Museum | Johnstonei A | M | Female | -15.99167 | 125.32833 | Prince Regent Nature Reserve |
| R171897 | Western Australian Museum | Johnstonei A | M | Female | -13.93972 | 126.17361 | Wargul Wargul Island |
| R171900 | Western Australian Museum | Johnstonei A | M | Male | -15.90944 | 124.46250 | Balami ridge |
| R172784 | Western Australian Museum | Johnstonei A | M | Female | -16.15889 | 125.30722 | King Leopold Ranges Conservation Park |
| R173481 | Western Australian Museum | Johnstonei A | SD | NA | -14.60824 | 126.93172 | Drysdale River |
| R117703 | Western Australian Museum | Johnstonei B | M | NA | -14.18750 | 125.73390 | Corneille Island |
| R117708 | Western Australian Museum | Johnstonei B | M | NA | -14.13910 | 125.69940 | Fenelon Island |
| R117709 | Western Australian Museum | Johnstonei B | M | NA | -14.13910 | 125.69940 | Fenelon Island |
| R117710 | Western Australian Museum | Johnstonei B | M | NA | -14.13860 | 125.69780 | Fenelon Island |
| R117751 | Western Australian Museum | Johnstonei B | M | NA | -14.18880 | 125.73220 | Corneille Island |
| R117945 | Western Australian Museum | Johnstonei B | M | Male | -14.18556 | 125.72444 | Corneille Island |
| R117953 | Western Australian Museum | Johnstonei B | SD/M | NA | -14.18580 | 125.73470 | Corneille Island |
| R117964 | Western Australian Museum | Johnstonei B | M | NA | -13.95160 | 125.64330 | Cassini Island |
| R117967 | Western Australian Museum | Johnstonei B | SD/M | NA | -14.18550 | 125.72440 | Corneille Island |
| R158508 | Western Australian Museum | Johnstonei B | M | NA | -14.28250 | 125.30470 | Don Island |
| R158540 | Western Australian Museum | Johnstonei B | M | Female | -14.29472 | 125.22556 | West Montalivet Island |
| R158543 | Western Australian Museum | Johnstonei B | M | Female | -14.29472 | 125.22556 | West Montalivet Island |
| R158546 | Western Australian Museum | Johnstonei B | M | Female | -14.29470 | 125.22560 | West Montalivet Island |
| R158561 | Western Australian Museum | Johnstonei B | M | Male | -14.29470 | 125.22560 | West Montalivet Island |
| R158562 | Western Australian Museum | Johnstonei B | M | Male | -14.29470 | 125.22560 | West Montalivet Island |
| R158564 | Western Australian Museum | Johnstonei B | M | Male | -14.29470 | 125.22560 | West Montalivet Island |
| R158565 | Western Australian Museum | Johnstonei B | M | Female | -14.29470 | 125.22560 | West Montalivet Island |
| R158569 | Western Australian Museum | Johnstonei B | M | Female | -14.29470 | 125.22560 | West Montalivet Island |
| R158571 | Western Australian Museum | Johnstonei B | M | Female | -14.29470 | 125.22560 | West Montalivet Island |
| R158572 | Western Australian Museum | Johnstonei B | M | Female | -14.29470 | 125.22560 | West Montalivet Island |
| R158573 | Western Australian Museum | Johnstonei B | M | Female | -14.29470 | 125.22560 | West Montalivet Island |
| R158580 | Western Australian Museum | Johnstonei B | M | Male | -14.27940 | 125.30610 | East Montalivet Island |
| R158581 | Western Australian Museum | Johnstonei B | M | Female | -14.27940 | 125.30610 | East Montalivet Island |
| R158582 | Western Australian Museum | Johnstonei B | M | Female | -14.27940 | 125.30610 | East Montalivet Island |
| R158583 | Western Australian Museum | Johnstonei B | SD/M | Male | -14.27944 | 125.30611 | East Montalivet Island |
| R158610 | Western Australian Museum | Johnstonei B | M | Female | -14.28250 | 125.30470 | Don Island |
| R158646 | Western Australian Museum | Johnstonei B | M | Female | -14.39830 | 124.97750 | North Maret Island |
| R158647 | Western Australian Museum | Johnstonei B | M | Female | -14.39830 | 124.97750 | North Maret Island |
| R158648 | Western Australian Museum | Johnstonei B | M | Female | -14.39830 | 124.97750 | North Maret Island |
| R158655 | Western Australian Museum | Johnstonei B | M | Female | -14.51660 | 124.98330 | Berthier Island |
| R158657 | Western Australian Museum | Johnstonei B | SD/M | NA | -14.51667 | 124.98333 | Berthier Island |
| R158661 | Western Australian Museum | Johnstonei B | M | Female | -14.51660 | 124.98330 | Berthier Island |
| R158683 | Western Australian Museum | Johnstonei B | M | NA | -14.39830 | 124.97750 | North Maret Island |
| R158705 | Western Australian Museum | Johnstonei B | SD/M | NA | -14.39833 | 124.97750 | North Maret Island |
| R165970 | Western Australian Museum | Johnstonei B | M | Female | -14.40139 | 124.97917 | Maret Island |
| ABTC11723/R34050 | South Australian Museum | Triacantha A | M | NA | -23.98333 | 120.01667 | Weelarrana |
| ABTC11847/R34151 | South Australian Museum | Triacantha A | M | NA | -12.65000 | 132.88333 | Jabiru |
| ABTC29091/R20877 | Northern Territory Museum | Triacantha A | SD | NA | -12.66667 | 132.88333 | Jabiru |
| ABTC29692/R22162 | South Australian Museum | Triacantha A | M | NA | -13.26528 | 130.96222 | Litchfield |
| ABTC33954/R42082 | South Australian Museum | Triacantha A | M | NA | -26.34972 | 130.17167 | Illintjitja |
| ABTC41790/R46088 | South Australian Museum | Triacantha A | SD/M | NA | -25.98028 | 129.46306 | Waltitjara |
| CCM1859 | Australian National University | Triacantha A | SD/M | Male | -23.58338 | 134.49942 | Ross River |
| CCM1860 | Australian National University | Triacantha A | M | Female | -23.58338 | 134.49942 | Ross River Dump |
| CCM1888 | Australian National University | Triacantha A | M | NA | -23.72534 | 133.34322 | Birthday Water Hole |
| R106160 | Western Australian Museum | Triacantha A | M | Male | -23.86667 | 118.56667 | Turee Creek |
| R108988 | Western Australian Museum | Triacantha A | M | NA | -20.26944 | 118.89722 | Lesley Salt Works |
| R108990 | Western Australian Museum | Triacantha A | M | NA | -20.28050 | 118.88060 | Lesley Salt Works |
| R110273 | Western Australian Museum | Triacantha A | M | NA | -22.82000 | 119.61250 | Mile Camp |
| R110905 | Western Australian Museum | Triacantha A | M | Male | -19.63222 | 128.87528 | Tanami Downs |
| R111810 | Western Australian Museum | Triacantha A | M | Female | -23.49861 | 120.29111 | Wheelarra Hill |
| R113038 | Western Australian Museum | Triacantha A | M | NA | -20.26944 | 118.89722 | Lesley Salt Works |
| R129921 | Western Australian Museum | Triacantha A | M | NA | -23.25000 | 118.66667 | West Angelas |
| R139010 | Western Australian Museum | Triacantha A | SD/M | NA | -19.80833 | 121.46389 | Mandora |
| R139404 | Western Australian Museum | Triacantha A | M | NA | -21.21639 | 120.34056 | Meentheena |
| R142456 | Western Australian Museum | Triacantha A | M | Male | -22.85056 | 127.83250 | Kiwirrkurra |
| R142973 | Western Australian Museum | Triacantha A | M | Female | -22.85050 | 127.83250 | Kiwirrkurra |
| R145500 | Western Australian Museum | Triacantha A | M | NA | -22.39000 | 119.99000 | Port Hedland |
| R145556 | Western Australian Museum | Triacantha A | M | NA | -20.91000 | 118.68000 | Port Hedland |
| R145752 | Western Australian Museum | Triacantha A | M | NA | -22.09000 | 118.99194 | Chichester Range |
| R154566 | Western Australian Museum | Triacantha A | M | NA | -23.37917 | 120.10528 | Newman |
| R157406 | Western Australian Museum | Triacantha A | M | Female | -19.58917 | 128.86028 | Tanami Desert |
| R157466 | Western Australian Museum | Triacantha A | M | Male | -19.89970 | 128.82700 | Tanami Desert |
| R158101 | Western Australian Museum | Triacantha A | M | NA | -21.60417 | 117.07750 | Millstream |
| R160269 | Western Australian Museum | Triacantha A | M | NA | -22.46639 | 119.02250 | Mount Marsh |
| R161603 | Western Australian Museum | Triacantha A | M | NA | -19.99833 | 119.35861 | Goldsworthy |
| R164546 | Western Australian Museum | Triacantha A | M | NA | -21.62194 | 116.38972 | Pannawonica |
| R170651 | Western Australian Museum | Triacantha A | M | NA | -21.62194 | 116.38972 | Pannawonica |
| ABTC28797/R16430 | Northern Territory Museum | Triacantha B | SD/M | NA | -18.60000 | 136.10000 | Brunette Downs |
| ABTC29531/R21401 | Northern Territory Museum | Triacantha B | SD/M | NA | -18.60833 | 137.98833 | Lawn Hill |
| ABTC29554/R21444 | South Australian Museum | Triacantha B | M | NA | -18.59583 | 138.13183 | Lawn Hill |
| ABTC29573/R21478 | South Australian Museum | Triacantha B | M | NA | -18.59583 | 138.13183 | Lawn Hill |
| ABTC29851/R22383 | Northern Territory Museum | Triacantha B | SD | NA | -15.97083 | 129.04028 | Keep River |
| CCM0738 | Australian National University | Triacantha B | M | NA | -15.19890 | 125.90350 | Spring Creek |
| CCM0888 | Australian National University | Triacantha B | M | Female | -14.83470 | 126.30013 | Theda Station |
| CCM0932 | Australian National University | Triacantha B | M | Female | -14.73298 | 126.46345 | Theda Station |
| CCM1130 | Australian National University | Triacantha B | SD/M | Female | -14.78110 | 126.63490 | Langoor Basalt |
| CCM1136 | Australian National University | Triacantha B | M | Female | -14.76990 | 126.57880 | Theda Station |
| CCM1199 | Australian National University | Triacantha B | M | Female | -14.83170 | 125.71920 | Mitchell Plateau Repeater Track |
| CCM1202 | Australian National University | Triacantha B | M | NA | -14.16710 | 125.73320 | Mitchell Plateau |
| CCM1235 | Australian National University | Triacantha B | M | NA | -17.04067 | 125.22680 | Bell Gorge |
| CCM1394 | Australian National University | Triacantha B | M | Male | -16.49954 | 125.33637 | Grevillea gorge |
| CCM1395 | Australian National University | Triacantha B | M | Male | -16.49954 | 125.33637 | Grevillea gorge |
| CCM1643 | Australian National University | Triacantha B | M | Female | -16.17108 | 125.98763 | Munja track |
| CCM1767 | Australian National University | Triacantha B | M | Female | -17.60560 | 126.04110 | Mornington Gorge |
| CCM1781 | Australian National University | Triacantha B | M | NA | -16.04730 | 126.70170 | Russ Creek |
| CCM1782 | Australian National University | Triacantha B | M | Male | -17.31000 | 126.07700 | Glenroy Meatworks |
| CCM1784 | Australian National University | Triacantha B | M | NA | -16.80000 | 125.84430 | Galvan Gorge |
| R108782 | Western Australian Museum | Triacantha B | M | NA | -17.25000 | 128.30000 | Bream Gorge |
| R114408 | Western Australian Museum | Triacantha B | M | NA | -16.08333 | 123.41667 | King Hall Island |
| R117796 | Western Australian Museum | Triacantha B | M | NA | -14.34194 | 126.02111 | Middle Osborn Island |
| R117798 | Western Australian Museum | Triacantha B | M | NA | -14.34330 | 126.02080 | Middle Osborn Island |
| R117853 | Western Australian Museum | Triacantha B | M | NA | -14.34917 | 125.95917 | South West Osborn Island |
| R117946 | Western Australian Museum | Triacantha B | M | NA | -14.13778 | 125.69861 | Fenelon Island |
| R146022 | Western Australian Museum | Triacantha B | M | NA | -16.68330 | 123.83330 | Kimbolton |
| R151868 | Western Australian Museum | Triacantha B | M | NA | -13.88333 | 126.56667 | Sir Graham Moore Island |
| R151958 | Western Australian Museum | Triacantha B | M | NA | -14.35000 | 125.95000 | South West Osborn Island |
| R158008 | Western Australian Museum | Triacantha B | M | Female | -16.14861 | 123.77944 | Koolan Island |
| R158016 | Western Australian Museum | Triacantha B | M | Female | -16.14580 | 123.74920 | Koolan Island |
| R158551 | Western Australian Museum | Triacantha B | M | Female | -14.29472 | 125.22556 | West Montalivet Island |
| R158609 | Western Australian Museum | Triacantha B | M | Female | -14.28250 | 125.30472 | Don Island |
| R158970 | Western Australian Museum | Triacantha B | M | Female | -16.14861 | 123.77944 | Koolan Island |
| R164310 | Western Australian Museum | Triacantha B | M | Female | -14.78333 | 125.03333 | Lamarck Island |
| R165402 | Western Australian Museum | Triacantha B | M | NA | -16.03444 | 123.53750 | Bathurst Island |
| R165555 | Western Australian Museum | Triacantha B | M | Male | -16.12220 | 123.73440 | Koolan Island |
| R166929 | Western Australian Museum | Triacantha B | M | Female | -14.82972 | 125.71889 | Mitchell Plateau |
| R168173 | Western Australian Museum | Triacantha B | M | Male | -15.07270 | 125.17970 | Boongaree Island |
| R168237 | Western Australian Museum | Triacantha B | M | NA | -16.07472 | 123.55000 | Irvine Island |
| R168392 | Western Australian Museum | Triacantha B | M | Female | -16.10500 | 123.51222 | Gibbings Island |
| R168394 | Western Australian Museum | Triacantha B | M | Female | -16.10500 | 123.51220 | Gibbings Island |
| R168407 | Western Australian Museum | Triacantha B | M | Male | -16.08360 | 123.54190 | Irvine Island |
| R168426 | Western Australian Museum | Triacantha B | M | Female | -16.14889 | 123.58833 | Margaret Island |
| R168462 | Western Australian Museum | Triacantha B | M | NA | -13.88333 | 126.56667 | Sir Graham Moore Island |
| R168590 | Western Australian Museum | Triacantha B | SD | NA | -14.98333 | 124.91667 | Coronation Island |
| R168675 | Western Australian Museum | Triacantha B | M | NA | -14.46667 | 125.53333 | Katers Island |
| R168707 | Western Australian Museum | Triacantha B | M | Male | -14.46660 | 125.53330 | Katers Island |
| R168762 | Western Australian Museum | Triacantha B | M | Female | -14.31667 | 126.00000 | Middle Osborn Island |
| R168910 | Western Australian Museum | Triacantha B | M | Female | -14.60000 | 125.11670 | Bigge Island |
| R169985 | Western Australian Museum | Triacantha B | M | Female | -16.19889 | 123.64583 | Yampi Peninsula |
| R169990 | Western Australian Museum | Triacantha B | M | Female | -16.17660 | 123.63940 | Yampi Peninsula |
| R171206 | Western Australian Museum | Triacantha B | M | Male | -15.35139 | 124.52694 | Augustus Island |
| R171211 | Western Australian Museum | Triacantha B | M | Male | -15.25972 | 124.44528 | Darcy Island |
| R171420 | Western Australian Museum | Triacantha B | M | Male | -15.98972 | 125.32944 | Prince Regent Nature Reserve |
| R171904 | Western Australian Museum | Triacantha B | M | NA | -16.62250 | 123.47111 | Lachlan Island |
| R171905 | Western Australian Museum | Triacantha B | M | Female | -13.93972 | 126.17361 | Wargul Wargul Island |
| R171906 | Western Australian Museum | Triacantha B | M | Female | -16.25639 | 123.82444 | Nw Molema Island |
| R171907 | Western Australian Museum | Triacantha B | M | Female | -16.55778 | 123.35472 | Long Island |
| R171908 | Western Australian Museum | Triacantha B | M | Female | -16.41306 | 123.17917 | Sunday Island |
| R171909 | Western Australian Museum | Triacantha B | M | Female | -15.90972 | 124.46278 | Balami ridge |
| R171912 | Western Australian Museum | Triacantha B | M | Female | -16.22250 | 123.44972 | Hidden Island |
| R171914 | Western Australian Museum | Triacantha B | M | NA | -16.55639 | 123.35528 | Long Island |
| R171916 | Western Australian Museum | Triacantha B | M | Female | -16.62361 | 123.47139 | Lachlan Island |
| R171918 | Western Australian Museum | Triacantha B | M | Male | -16.25300 | 123.82280 | Nw Molema Island |
| R171919 | Western Australian Museum | Triacantha B | M | NA | -15.91083 | 124.46056 | Balami ridge |
| R171921 | Western Australian Museum | Triacantha B | M | Female | -15.94944 | 124.55944 | Storr Island |
| R171928 | Western Australian Museum | Triacantha B | M | NA | -15.90944 | 124.46250 | Balami ridge |
| R171930 | Western Australian Museum | Triacantha B | M | NA | -15.91194 | 124.46250 | Balami ridge |
| R171933 | Western Australian Museum | Triacantha B | M | Male | -15.91028 | 124.46139 | Balami ridge |
| ABTC61613 | South Australian Museum | Triacantha B* | SD | NA | -15.00500 | 129.58330 | Victoria River |
|  |  |  |  |  |  |  |  |
|  |  |  |  |  |  |  |  |
