# Supplementary material for: Validation and description of two new north-western Australian Rainbow skinks with multispecies coalescent methods and morphology
Source: PeerJ. 2017 Aug 29;5:e3724. doi: 10.7717/peerj.3724 (PMC5580384; doi:10.7717/peerj.3724)
Supplement: Table S2 — jModelTest substitution models used with the two StarBeast2 datasets and fragment length of loci. Loci designation based on Anolis carolinensis genome and sequence size that was retrieved for all used samples for each locus. [file peerj-05-3724-s002.docx]

| **Supplemental Table S2** – jModelTest substitution models used with the two StarBeast2 | | | |
| --- | --- | --- | --- |
| datasets and fragment length of loci. Loci designation based on *Anolis carolinensis* | | | |
| genome and sequence size that was retrieved for all used samples for each locus. | | | |
| Loci | Best jmodelTest model | Sequence size | Dataset |
| ENSACAP00000000634_exon4 | K80+G | 972 | StarBeast2 gene set1 |
| ENSACAP00000001488_exon1 | K80 | 330 | StarBeast2 gene set1 |
| ENSACAP00000001542_exon1 | K80 | 378 | StarBeast2 gene set1 |
| ENSACAP00000001948_exon6 | HKY | 954 | StarBeast2 gene set1 |
| ENSACAP00000002281_exon14 | HKY+G | 612 | StarBeast2 gene set1 |
| ENSACAP00000003365_exon1 | K80+G | 684 | StarBeast2 gene set1 |
| ENSACAP00000003748_exon1 | F81 | 624 | StarBeast2 gene set1 |
| ENSACAP00000003779_exon1 | K80 | 672 | StarBeast2 gene set1 |
| ENSACAP00000004256_exon6 | HKY | 534 | StarBeast2 gene set1 |
| ENSACAP00000005126_exon23 | K80 | 456 | StarBeast2 gene set1 |
| ENSACAP00000005482_exon1 | HKY+G | 432 | StarBeast2 gene set1 |
| ENSACAP00000006894_exon1 | HKY | 900 | StarBeast2 gene set1 |
| ENSACAP00000008371_exon1 | HKY | 318 | StarBeast2 gene set1 |
| ENSACAP00000012201_exon13 | HKY+G | 930 | StarBeast2 gene set1 |
| ENSACAP00000013421_exon9 | HKY | 354 | StarBeast2 gene set1 |
| ENSACAP00000014062_exon1 | K80+G | 474 | StarBeast2 gene set1 |
| ENSACAP00000014986_exon1 | JC | 588 | StarBeast2 gene set1 |
| ENSACAP00000016468_exon2 | K80 | 516 | StarBeast2 gene set1 |
| ENSACAP00000016542_exon10 | HKY | 360 | StarBeast2 gene set1 |
| ENSACAP00000016846_exon5 | HKY+G | 945 | StarBeast2 gene set1 |
| ENSACAP00000000100_exon1 | K80 | 534 | StarBeast2 gene set2 |
| ENSACAP00000001732_exon2 | K80+G | 810 | StarBeast2 gene set2 |
| ENSACAP00000001814_exon1 | K80 | 312 | StarBeast2 gene set2 |
| ENSACAP00000001953_exon1 | K80 | 624 | StarBeast2 gene set2 |
| ENSACAP00000002002_exon1 | HKY | 624 | StarBeast2 gene set2 |
| ENSACAP00000002448_exon12 | K80+G | 378 | StarBeast2 gene set2 |
| ENSACAP00000002929_exon1 | HKY+G | 480 | StarBeast2 gene set2 |
| ENSACAP00000003301_exon2 | K80 | 366 | StarBeast2 gene set2 |
| ENSACAP00000003869_exon1 | K80 | 528 | StarBeast2 gene set2 |
| ENSACAP00000004927_exon21 | HKY+G | 702 | StarBeast2 gene set2 |
| ENSACAP00000005547_exon1 | K80 | 438 | StarBeast2 gene set2 |
| ENSACAP00000008152_exon1 | K80 | 396 | StarBeast2 gene set2 |
| ENSACAP00000008956_exon1 | HKY | 576 | StarBeast2 gene set2 |
| ENSACAP00000009963_exon1 | K80 | 522 | StarBeast2 gene set2 |
| ENSACAP00000011288_exon1 | K80+G | 564 | StarBeast2 gene set2 |
| ENSACAP00000011827_exon1 | K80 | 504 | StarBeast2 gene set2 |
| ENSACAP00000012422_exon5 | K80+G | 588 | StarBeast2 gene set2 |
| ENSACAP00000012909_exon8 | K80 | 300 | StarBeast2 gene set2 |
| ENSACAP00000019243_exon1 | JC | 480 | StarBeast2 gene set2 |
| ENSACAP00000019346_exon9 | K80 | 489 | StarBeast2 gene set2 |
