# Supplementary material for: Validation and description of two new north-western Australian Rainbow skinks with multispecies coalescent methods and morphology
Source: PeerJ. 2017 Aug 29;5:e3724. doi: 10.7717/peerj.3724 (PMC5580384; doi:10.7717/peerj.3724)
Supplement: Table S3 [file peerj-05-3724-s003.docx]

| **Supplemental Table S3 –** Priors used for Starbeast2 and SNAPP analyses. | | | |
| --- | --- | --- | --- |
| **StarBeast2 priors** | | | |
|  | Lower limit | Upper limit | Distribution |
| Speciation rate | 10 | 1000 | 1/X |
| Clock rates | - | - | Lognormal (real-space mean = 1, sd = 1) |
| Population mean | 0.01 | 0.0001 | 1/X |
| HKY kappa (I.I.D.) | - | - | Lognormal (log-space mean = 1, sd = 1.25) |
| Gamma rates (I.I.D.) | - | - | Exponential (rate = 1) |
| **SNAPP priors** | | | |
|  | Lower limit | Upper limit | Distribution |
| Speciation rate | 1 | 100 | Uniform |
| Population thetas (I.I.D.) | - | - | Gamma (scale = beta, shape = 2) |
| Beta | 20 | 200 | Uniform |
