# Supplementary material for: Validation and description of two new north-western Australian Rainbow skinks with multispecies coalescent methods and morphology
Source: PeerJ. 2017 Aug 29;5:e3724. doi: 10.7717/peerj.3724 (PMC5580384; doi:10.7717/peerj.3724)
Supplement: Table S4 — The results for testing normality and heteroscedasticity are also presented for both the log-transformed and the log and size-corrected dataset. Bold are significant p-values for the MANOVA results. After removing samples with missing data, analyses were performed with a total of 92 specimens. [file peerj-05-3724-s004.docx]

**Supplemental Table S4 –** Summary of MANOVA results testing for significant interaction with mtDNA lineage within *C. johnstonei*. The results for testing normality and heteroscedasticity are also presented for both the log-transformed and the log and size-corrected dataset. Bold are significant *p-values* for the MANOVA results. After removing samples with missing data, analyses were performed with a total of 92 specimens.

|  | **Log transformed** | | | | **Log and size corrected** | | | |
| --- | --- | --- | --- | --- | --- | --- | --- | --- |
|  | **Shapiro-Wilk Test** | **Levene’s Test** | **F value** | ***p-value*** | **Shapiro-Wilk Test** | **Levene's Test** | **F value** | ***p-value*** |
| **Snout vent length** | 0.48 | 0.06 | 27.47 | **1.05E-06** |  |  |  |  |
| **Axilla to groin length** | 0.41 | 0.21 | 23.62 | **4.93E-06** | 0.38 | 0.50 | 0.05 | 8.21E-01 |
| **Head length** | 0.10 | 0.08 | 25.23 | **2.56E-06** | 0.35 | 0.27 | 0.83 | 3.66E-01 |
| **Head width** | 0.26 | 0.25 | 23.08 | **6.18E-06** | 0.32 | 0.96 | 0.32 | 5.71E-01 |
| **Head depth** | 0.06 | 0.18 | 45.57 | **1.40E-09** | 0.10 | 0.28 | 10.93 | **1.36E-03** |
| **Forelimb length** | 0.63 | 0.63 | 38.95 | **1.40E-08** | 0.39 | 0.60 | 7.39 | **7.89E-03** |
| **Hindlimb length** | 0.26 | 0.14 | 34.76 | **6.42E-08** | 0.11 | 1.00 | 5.16 | **2.55E-02** |
| **Nasals separation** | 0.16 | 0.15 | 6.04 | **1.59E-02** | 0.50 | 0.15 | 7.12 | **9.02E-03** |
| **Ear aperture length** | 0.33 | 0.98 | 20.63 | **1.72E-05** | 1.82E-03 | 0.93 | 2.63 | 1.09E-01 |
| **Palpebral distance length** | 0.17 | 0.06 | 10.10 | **2.03E-03** | 0.30 | 0.07 | 0.01 | 9.06E-01 |
| **Eye to ear distance** | 0.09 | 1.51E-03 | 32.06 | **1.76E-07** | 0.37 | 0.09 | 3.32 | 7.17E-02 |
| **MANOVA** | | | 8.37 | **1.37E-09** |  |  | 3.49 | **7.35E-04** |
