# Supplementary material for: Validation and description of two new north-western Australian Rainbow skinks with multispecies coalescent methods and morphology
Source: PeerJ. 2017 Aug 29;5:e3724. doi: 10.7717/peerj.3724 (PMC5580384; doi:10.7717/peerj.3724)
Supplement: Table S5 — The results for testing normality and heteroscedasticity are also presented for both the log-transformed and the log and size-corrected dataset. Bold are significant p-values for the MANOVA results. ∗, non-normal variables with significant support with the non-parametric Wilcoxon test. After removing samples with missing data, analyses were performed with a total of 94 specimens. [file peerj-05-3724-s005.docx]

**Supplemental Table S5 –** Summary of MANOVA results testing for significant interaction with mtDNA lineage within *C. triacantha*. The results for testing normality and heteroscedasticity are also presented for both the log-transformed and the log and size-corrected dataset. Bold are significant *p-values* for the MANOVA results. * - non-normal variables with significant support with the non-parametric Wilcoxon test. After removing samples with missing data, analyses were performed with a total of 94 specimens.

|  | **Log transformed** | | | | **Log and size corrected** | | | |
| --- | --- | --- | --- | --- | --- | --- | --- | --- |
|  | **Shapiro-Wilk Test** | **Levene's Test** | **F value** | ***p-value*** | **Shapiro-Wilk Test** | **Levene's Test** | **F value** | ***p-value*** |
| **Snout vent length** | 3.75E-05 | 0.90 | 7.62 | **0.00696*** |  |  |  |  |
| **Axilla to groin length** | 1.38E-04 | 0.57 | 5.93 | **1.68E-02** | 0.24 | 0.54 | 0.30 | 5.87E-01 |
| **Head length** | 3.63E-03 | 0.87 | 19.34 | **2.94E-05** | 0.02 | 0.33 | 13.92 | **0.00033*** |
| **Head width** | 2.35E-03 | 0.76 | 7.89 | **6.09E-03** | 0.16 | 0.06 | 0.51 | 4.78E-01 |
| **Head depth** | 1.94E-05 | 0.96 | 4.10 | **4.58E-02** | 0.04 | 0.81 | 0.53 | 4.68E-01 |
| **Forelimb length** | 0.08 | 0.09 | 10.76 | **1.46E-03** | 0.24 | 0.61 | 2.80 | 9.75E-02 |
| **Hindlimb length** | 5.65E-06 | 0.40 | 6.70 | **1.12E-02** | 5.93E-05 | 5.22E-03 | 0.31 | 5.79E-01 |
| **Nasals separation** | 0.05 | 0.47 | 1.64 | 2.04E-01 | 0.91 | 0.65 | 3.23 | 7.55E-02 |
| **Ear aperture length** | 0.16 | 0.33 | 9.99 | **2.14E-03** | 0.32 | 0.56 | 2.56 | 1.13E-01 |
| **Palpebral distance length** | 2.58E-03 | 0.84 | 2.97 | 8.84E-02 | 5.69E-06 | 0.65 | 4.00E-03 | 9.48E-01 |
| **Eye to ear distance** | 4.10E-03 | 0.60 | 13.41 | **4.18E-04** | 0.03 | 0.83 | 5.03 | **0.0273*** |
| **Manova** | | | 4.92 | **8.07E-06** |  |  | 3.69 | **2.75E-04** |
