# Supplementary material for: Validation and description of two new north-western Australian Rainbow skinks with multispecies coalescent methods and morphology
Source: PeerJ. 2017 Aug 29;5:e3724. doi: 10.7717/peerj.3724 (PMC5580384; doi:10.7717/peerj.3724)
Supplement: Table S6 — Summary of Generalized Linear modelling with a Poisson distribution analyses for relevant meristic variables, presenting estimates and respective confidence intervals (C.I.). Bold correspond to significant p-values. After removing samples with missing data, analyses were performed with a total of 85 and 83 specimens for C. johnstonei and C. triacantha, respectively. [file peerj-05-3724-s006.docx]

**Supplemental Table S6 –** Summary of Generalized Linear modelling with a Poisson distribution analyses for relevant meristic variables, presenting estimates and respective confidence intervals (C.I.). Bold correspond to significant *p-values.* After removing samples with missing data, analyses were performed with a total of 85 and 83 specimens for *C. johnstonei* and *C. triacantha*, respectively.

|  |  | **Estimate** | **C.I.** | **Std. Error** | **z value** | ***p-value*** |
| --- | --- | --- | --- | --- | --- | --- |
| *C. johnstonei* | **Lamellae under the 3rd finger** | 0.162 | 0.065-0.257 | 0.049 | 3.302 | **9.60E-04** |
|  | **Lamellae under the 4th toe** | 0.142 | 0.059-0.225 | 0.042 | 3.370 | **7.51E-04** |
|  | **Ear lobules number** | 0.319 | 0.198-0.439 | 0.062 | 5.177 | **2.25E-07** |
| *C. triacantha* | **Lamellae under the 3rd finger** | 0.020 | -0.083-0.125 | 0.053 | 0.378 | 0.705 |
|  | **Lamellae under the 4th toe** | -0.006 | -0.096-0.086 | 0.046 | -0.121 | 0.904 |
|  | **Ear lobules number** | 0.412 | 0.238-0.592 | 0.090 | 4.555 | **5.24E-06** |
