# Supplementary material for: Validation and description of two new north-western Australian Rainbow skinks with multispecies coalescent methods and morphology
Source: PeerJ. 2017 Aug 29;5:e3724. doi: 10.7717/peerj.3724 (PMC5580384; doi:10.7717/peerj.3724)
Supplement: Table S7 [file peerj-05-3724-s007.docx]

**Supplemental Table S7 –** Descriptive table with measurements and meristic data for each main lineage within *C. johnstonei* and *C. triacantha*.

|  | **Johnstonei A** | **Johnstonei B** | **Triacantha A** | **Triacantha B** |
| --- | --- | --- | --- | --- |
| **Snout vent length** | 36.39 (66) | 41.83 (35) | 36.55 (31) | 40.07 (68) |
|  | (21.84 - 43.49) | (27.93 - 51.44) | (23.78 - 44.98) | (24.72 - 49.12) |
| **Axilla to groin length** | 16.31 (66) | 19.13 (35) | 16.12 (31) | 17.62 (68) |
|  | (8.53 - 23.86) | (11.45 - 24.37) | (9.56 - 21.53) | (10.77 - 24.44) |
| **Head length** | 7.63 (66) | 8.67 (35) | 7.24 (31) | 8.25 (68) |
|  | (4.88 - 9.8) | (5.57 - 10.61) | (5.17 - 9.14) | (5.34 - 10.1) |
| **Head width** | 5.45 (66) | 6.17 (35) | 5.53 (31) | 6 (68) |
|  | (3.7 - 7.04) | (4.27 - 7.92) | (4.06 - 6.89) | (4.04 - 7.64) |
| **Head depth** | 3.59 (66) | 4.48 (35) | 3.57 (31) | 3.81 (68) |
|  | (1.81 - 4.49) | (2.48 - 6.07) | (1.59 - 4.34) | (2.4 - 4.86) |
| **Nasals separation** | 2.04 (64) | 2.19 (33) | 2.16 (30) | 2.23 (68) |
|  | (1.43 - 2.46) | (1.66 - 2.67) | (1.67 - 2.75) | (1.55 - 2.71) |
| **Ear aperture length** | 1.01 (62) | 1.27 (32) | 1.13 (30) | 1.33 (67) |
|  | (0.50 - 1.44) | (0.85 - 2.16) | (0.64 - 1.73) | (0.69 - 1.96) |
| **Palpebral disc length** | 1.31 (62) | 1.44 (32) | 1.41 (30) | 1.46 (67) |
|  | (1.05 - 1.59) | (1.04 - 2.13) | (0.99 - 1.71) | (0.92 - 1.81) |
| **Eye to ear distance** | 2.66 (62) | 3.26 (32) | 2.41 (30) | 2.75 (67) |
|  | (1.79 - 3.51) | (2.10 - 4.4) | (1.55 - 2.90) | (1.47 - 3.61) |
| **Forelimb length** | 9.51 (63) | 11.45 (33) | 10.49 (29) | 11.53 (67) |
|  | (6.17 - 14.14) | (8.60 - 13.94) | (7.94 - 12.27) | (7.27 - 14.54) |
| **Hindlimb length** | 14.82 (63) | 17.77 (33) | 16.74 (29) | 18.08 (68) |
|  | (8.80 - 20.12) | (12.67 - 23.18) | (10.73 - 24.01) | (12.37 - 21.21) |
| **Lamellae under the 3rd finger** | 16.75 (63) | 19.69 (35) | 18.83 (30) | 19.27 (62) |
|  | (9 - 20) | (17 - 23) | (16 - 22) | (11 - 24) |
| **Lamellae under the 4th toe** | 22.83 (63) | 26.31 (35) | 24.83 (29) | 24.82 (62) |
|  | (15 - 27) | (21 - 30) | (23 - 28) | (18 - 30) |
| **Ear lobules number** | 9.68 (63) | 13.31 (35) | 5.86 (28) | 8.81 (62) |
|  | (5 - 16) | (8 - 18) | (1 - 8) | (1 - 14) |
| **Number of keels** | 2 (63) | 2.34 (35) | 3 (30) | 3 (63) |
|  | (2 - 2) | (2 - 3) | (3 - 3) | (3 - 3) |
| **Number of supraciliary scales** | 6.94 (63) | 6.94 (35) | 6.07 (30) | 5.79 (63) |
|  | (6 - 8) | (5 - 8) | (5 - 7) | (5 - 7) |
| **Number of Supralabial scales** | 7.03 (63) | 7.14 (35) | 7.1 (30) | 7 (63) |
|  | (7 - 8) | (7 - 8) | (7 - 8) | (6 - 8) |
| **Number of infralabial scales** | 6.27 (63) | 6.11 (35) | 6.3 (30) | 6.06 (63) |
|  | (6 - 7) | (6 - 7) | (6 - 7) | (5 - 7) |
