# Supplementary material for: Validation and description of two new north-western Australian Rainbow skinks with multispecies coalescent methods and morphology
Source: PeerJ. 2017 Aug 29;5:e3724. doi: 10.7717/peerj.3724 (PMC5580384; doi:10.7717/peerj.3724)
Supplement: Figure S1 — mtDNA ND4 maximum likelihood phylogenetic tree of Carlia triacantha and Carlia johnstonei, from Afonso Silva et al. (2017), with specimens that were analysed by morphological analyses. Sample label includes tissue number, original ID and sampling location. [file peerj-05-3724-s008.pdf]

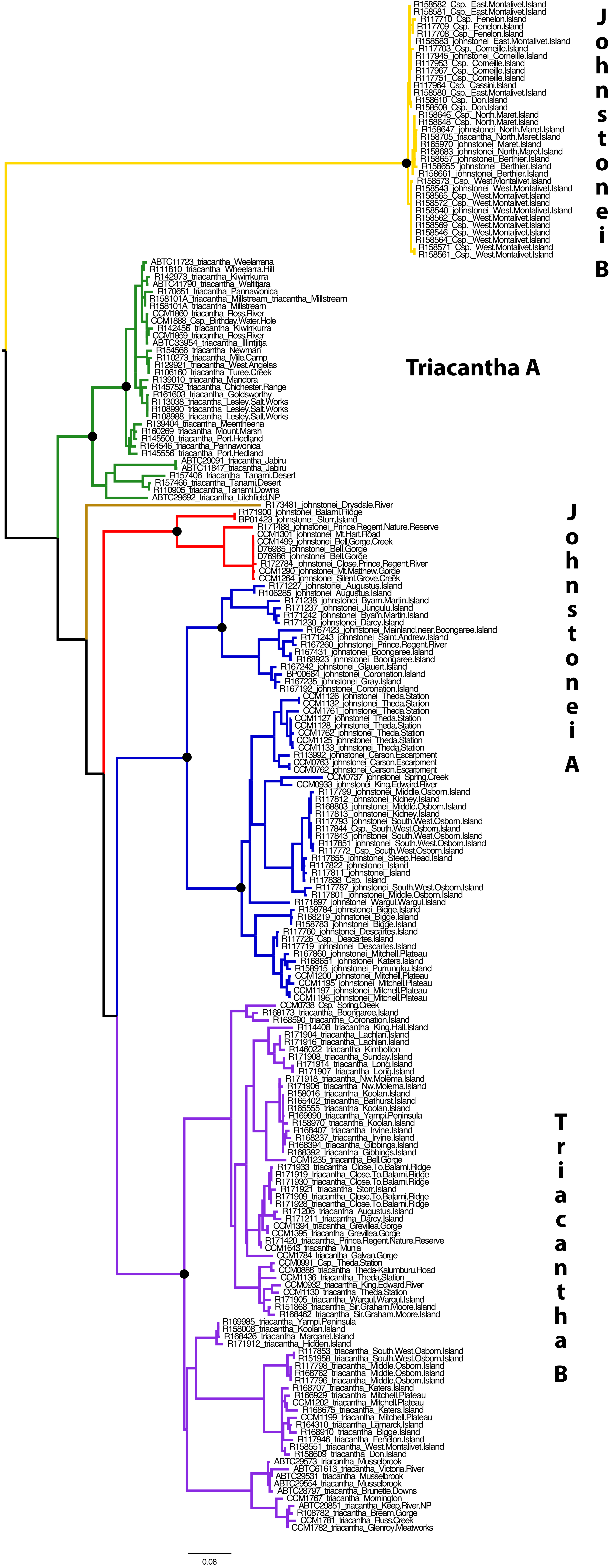

Supplemental Figure S1 – mtDNA ND4 maximum likelihood phylogenetic tree of *Carlia triacantha* and *Carlia johnstonei*, from Afonso Silva et al. (2017), with specimens that were analysed by morphological analyses. Sample label includes tissue number, original ID and sampling location.
