# Supplementary material for: Validation and description of two new north-western Australian Rainbow skinks with multispecies coalescent methods and morphology
Source: PeerJ. 2017 Aug 29;5:e3724. doi: 10.7717/peerj.3724 (PMC5580384; doi:10.7717/peerj.3724)
Supplement: Figure S2 — This method calculates the marginal likelihoods from the area under the likelihood posterior curve. Two replicate chains were run for each method and dataset. The mean likelihoods are plotted with + symbols for one chain, and × symbols for the other. Segmented lines approximating the curve are plotted by connecting the likelihoods averaged for both chains. [file peerj-05-3724-s009.pdf]

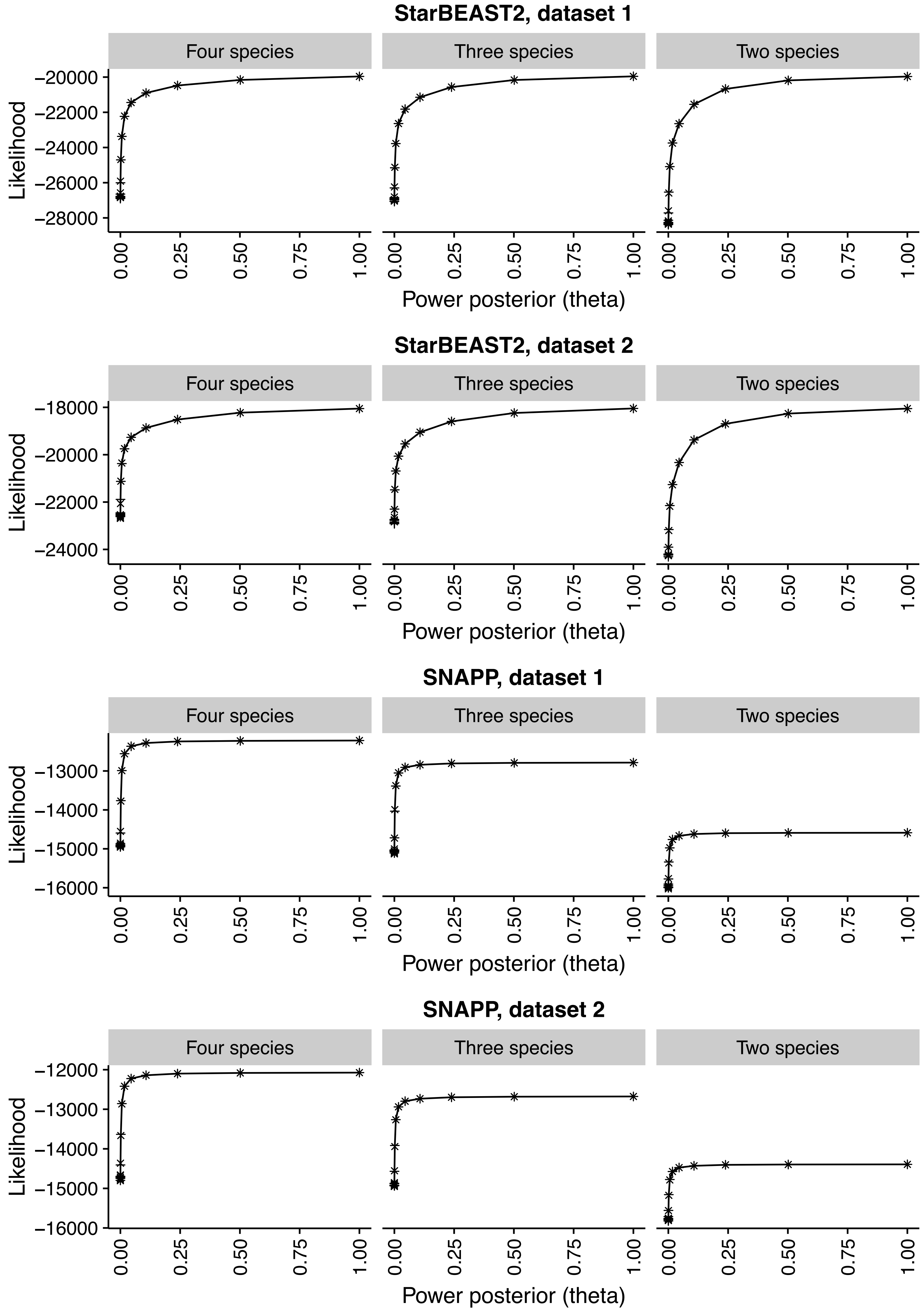

Supplemental Figure S2 - Stepping-stone computation of marginal likelihoods for Bayes factor species delimitation. This method calculates the marginal likelihoods from the area under the likelihood posterior curve. Two replicate chains were run for each method and dataset. The mean likelihoods are plotted with + symbols for one chain, and x symbols for the other. Segmented lines approximating the curve are plotted by connecting the likelihoods averaged for both chains.
