# Supplementary material for: Validation and description of two new north-western Australian Rainbow skinks with multispecies coalescent methods and morphology
Source: PeerJ. 2017 Aug 29;5:e3724. doi: 10.7717/peerj.3724 (PMC5580384; doi:10.7717/peerj.3724)
Supplement: Figure S3 — (A) Lateral view of specimen with ear aperture length (EAL), eye to ear distance (EED) and palpebral disc length (PDL). (C) Nasal separation (NS) measured in dorsal view. (C) and (D) correspond to forelimb (FLL) and hindlimb length (HLL) measurements in ventral view. Photos by Damien Esquerré. [file peerj-05-3724-s010.pdf]

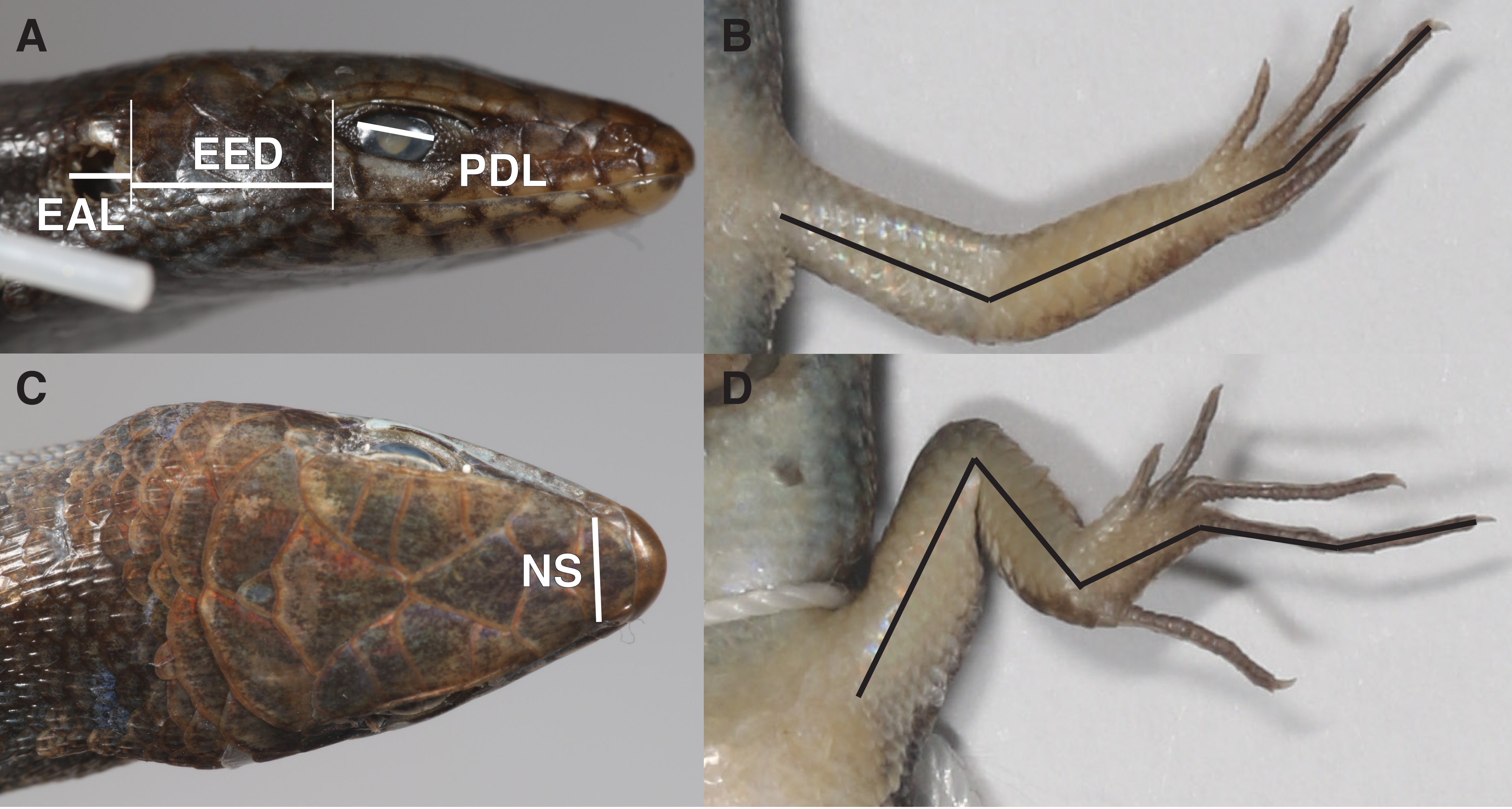

Supplemental Figure S3 – Measurements taken from photos.
A) Lateral view of specimen with ear aperture length (EAL), eye to ear distance (EED) and palpebral disc length (PDL). C) Nasal separation (NS) measured in dorsal view. C) and D) correspond to forelimb (FLL) and hindlimb length (HLL) measurements in ventral view. Photos by Damien Esquerré.
