# Supplementary material for: Validation and description of two new north-western Australian Rainbow skinks with multispecies coalescent methods and morphology
Source: PeerJ. 2017 Aug 29;5:e3724. doi: 10.7717/peerj.3724 (PMC5580384; doi:10.7717/peerj.3724)
Supplement: Figure S4 — JA, Johnstonei A; JB, Johnstonei B; TA, Triacantha A; TB, Triacantha B. [file peerj-05-3724-s011.pdf]

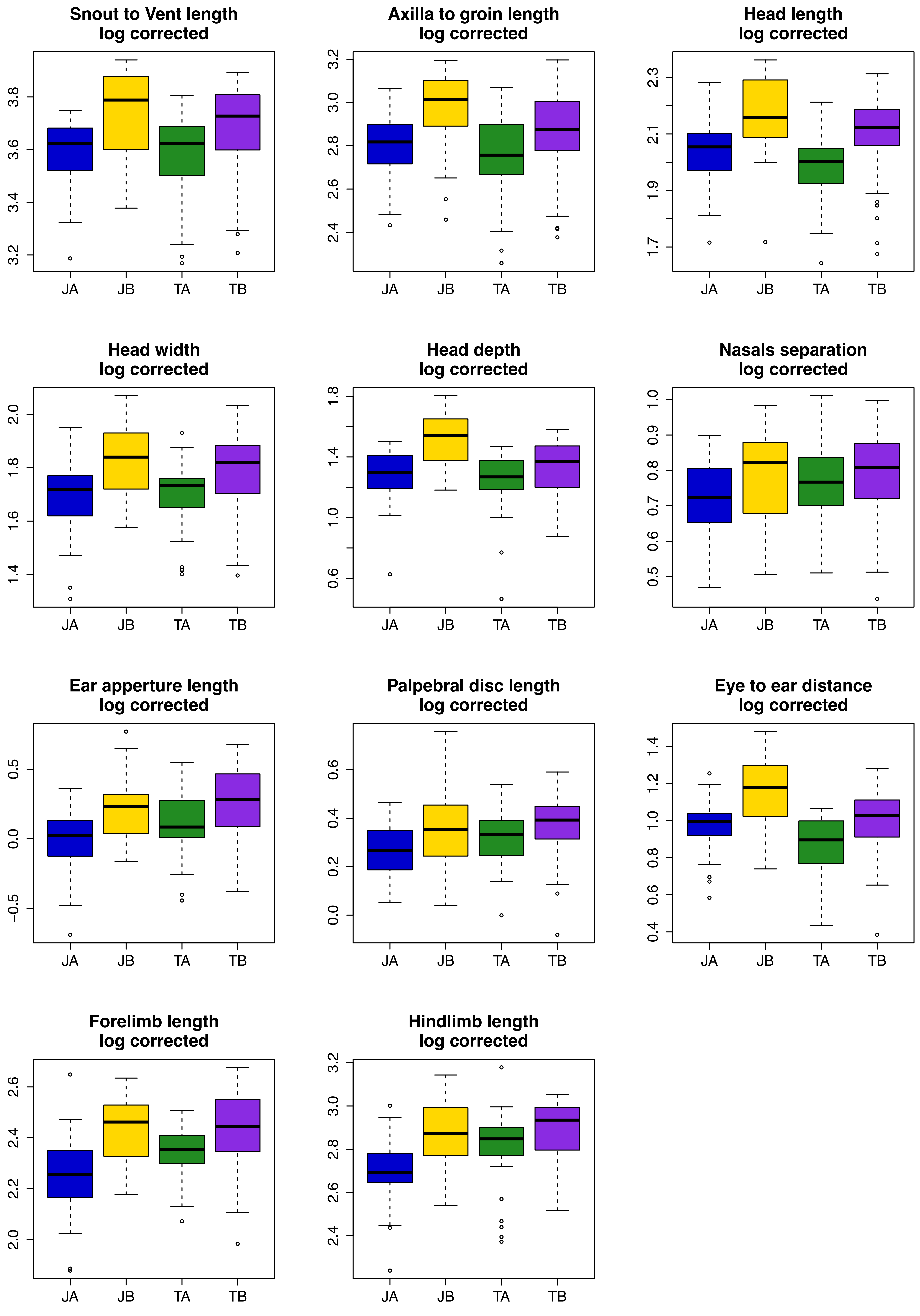

Supplemental Figure S4 – Box plots for all log transformed variables. JA – Johnstonei A, JB – Johnstonei B, TA – Triacantha A, TB – Triacantha B.
