# Supplementary material for: Validation and description of two new north-western Australian Rainbow skinks with multispecies coalescent methods and morphology
Source: PeerJ. 2017 Aug 29;5:e3724. doi: 10.7717/peerj.3724 (PMC5580384; doi:10.7717/peerj.3724)
Supplement: Figure S6 — PCA loadings and variables importance of PCA with log transformed data (A, B) and with size corrected data (C, D) for C. johnstonei and C. triacantha. [file peerj-05-3724-s013.pdf]

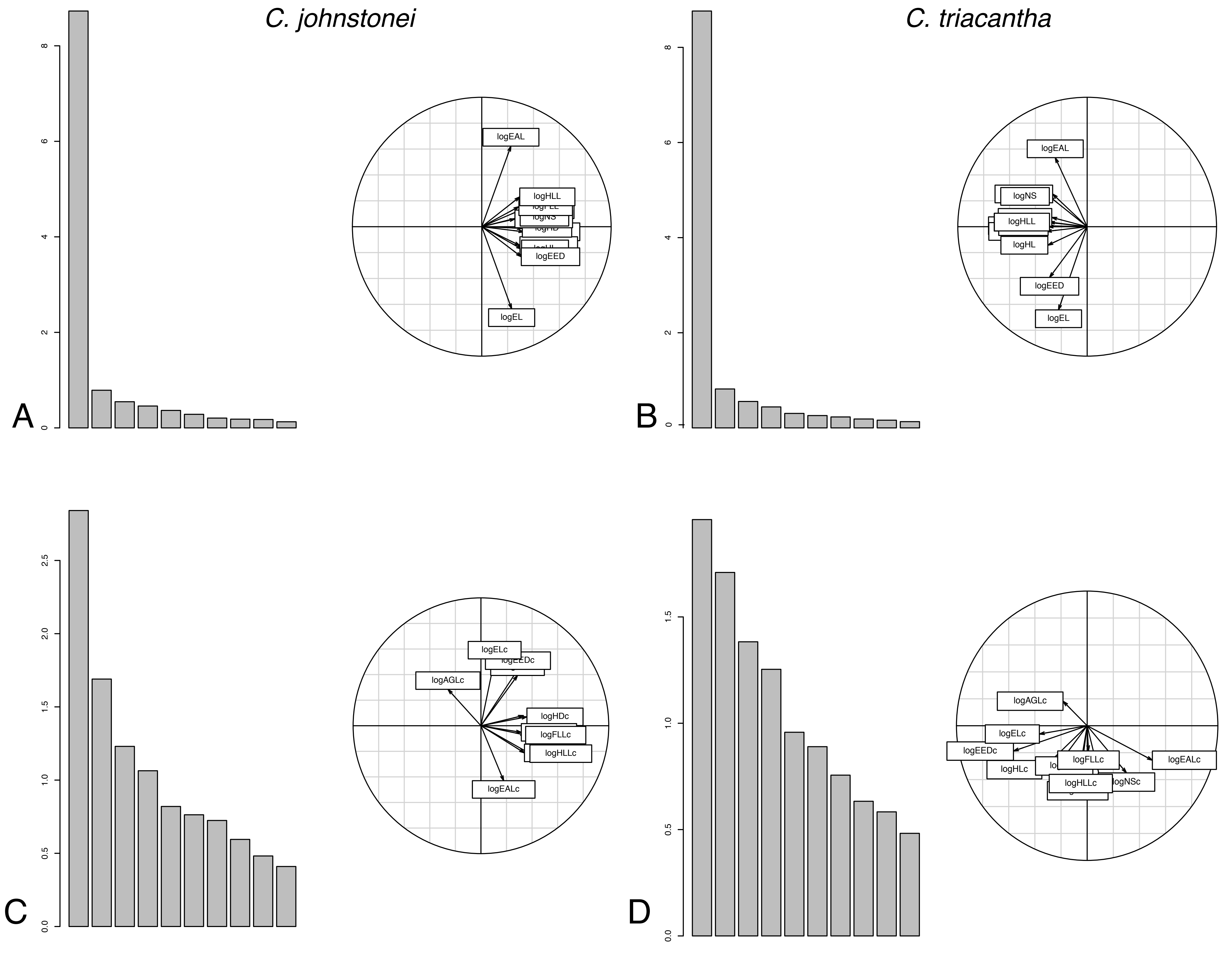

Supplemental Figure S6 – PCA loadings and variables importance of PCA with log transformed data (A, B) and with size corrected data (C, D) for *C. johnstonei* and *C. triacantha*.
