# Supplementary material for: Validation and description of two new north-western Australian Rainbow skinks with multispecies coalescent methods and morphology
Source: PeerJ. 2017 Aug 29;5:e3724. doi: 10.7717/peerj.3724 (PMC5580384; doi:10.7717/peerj.3724)
Supplement: Figure S7 — Dorsal and ventral view of holotypes of C. insularis sp. nov. (A), specimen WAM R158646, and C. isostriacantha sp. nov. (B), specimen WAM R171420. All photos by Damien Esquerré. [file peerj-05-3724-s014.pdf]

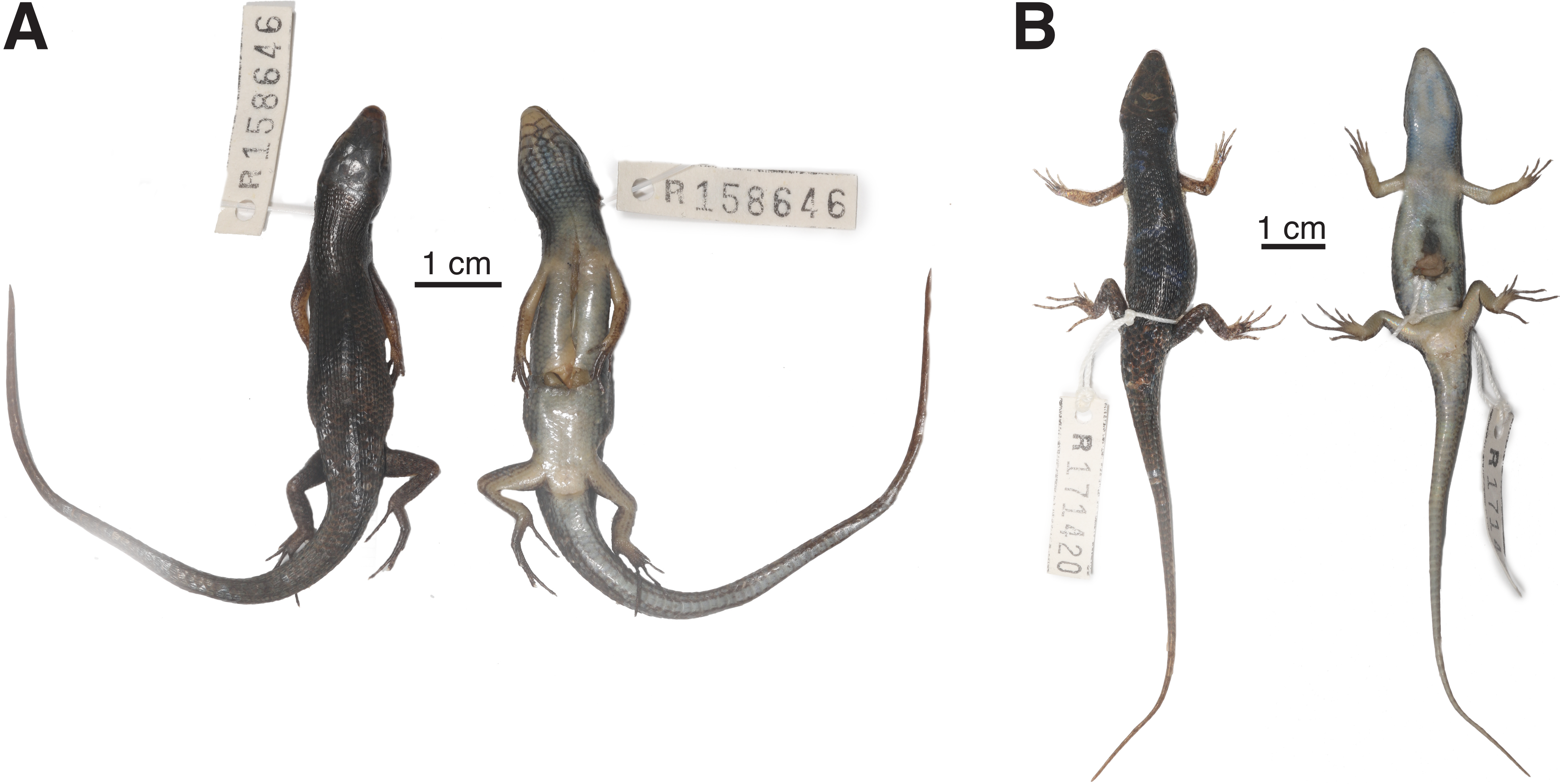

Supplemental Figure S7 – Dorsal and ventral view of holotypes of *C. insularis* sp. nov. (A), specimen WAM R158646, and *C. isostriacantha* sp. nov. (B), specimen WAM R171420. All photos by Damien Esquerré.
