# Supplementary material for: Validation and description of two new north-western Australian Rainbow skinks with multispecies coalescent methods and morphology
Source: PeerJ. 2017 Aug 29;5:e3724. doi: 10.7717/peerj.3724 (PMC5580384; doi:10.7717/peerj.3724)
Supplement: Figure S9 — Photos with live animals showing breeding colours of C. insularis sp. nov. (A, photo by Russell Barrett) and a potential diagnostic trait in C. isostriacantha sp. nov. (B, photo by Mark Allen). The white arrow points to the potential white line trait that distinguish this species from C. triacantha. [file peerj-05-3724-s016.pdf]

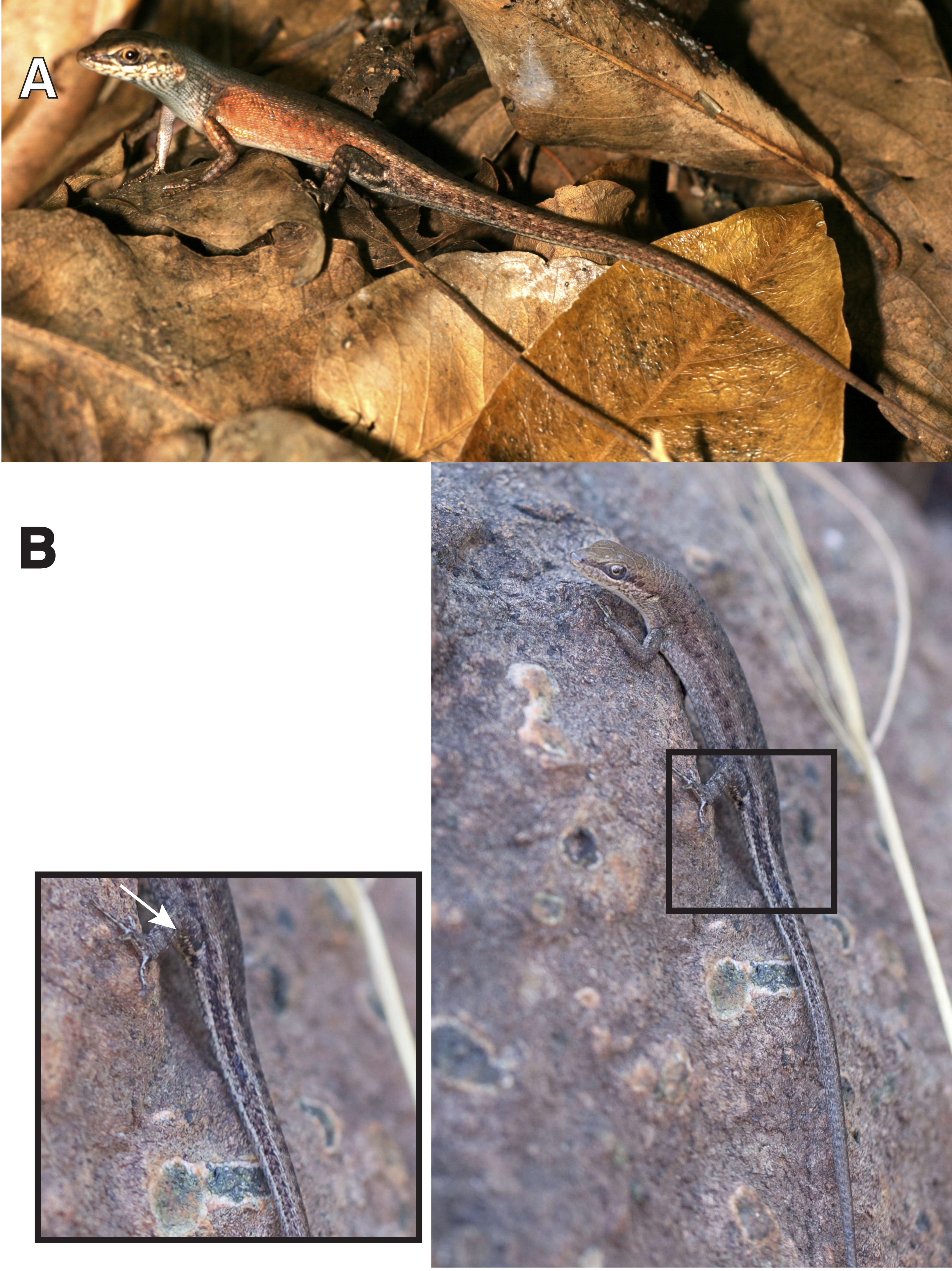

Supplemental Figure S9 – Photos with live animals showing breeding colours of *C. insularis* sp. nov. (A, photo by Russell Barrett) and a potential diagnostic trait in *C. isostriacantha* sp. nov. (B, photo by Mark Allen). The white arrow points to the potential white line trait that distinguish this species from *C. triacantha*.
