# Supplementary material for: Validation and description of two new north-western Australian Rainbow skinks with multispecies coalescent methods and morphology
Source: PeerJ. 2017 Aug 29;5:e3724. doi: 10.7717/peerj.3724 (PMC5580384; doi:10.7717/peerj.3724)
Supplement: Figure S8 — Dorsal and ventral paratype photos for C. insularis sp. nov (A, C) and for C. isostriacantha sp. nov. (B, D). All photos by Damien Esquerré. [file peerj-05-3724-s015.pdf]

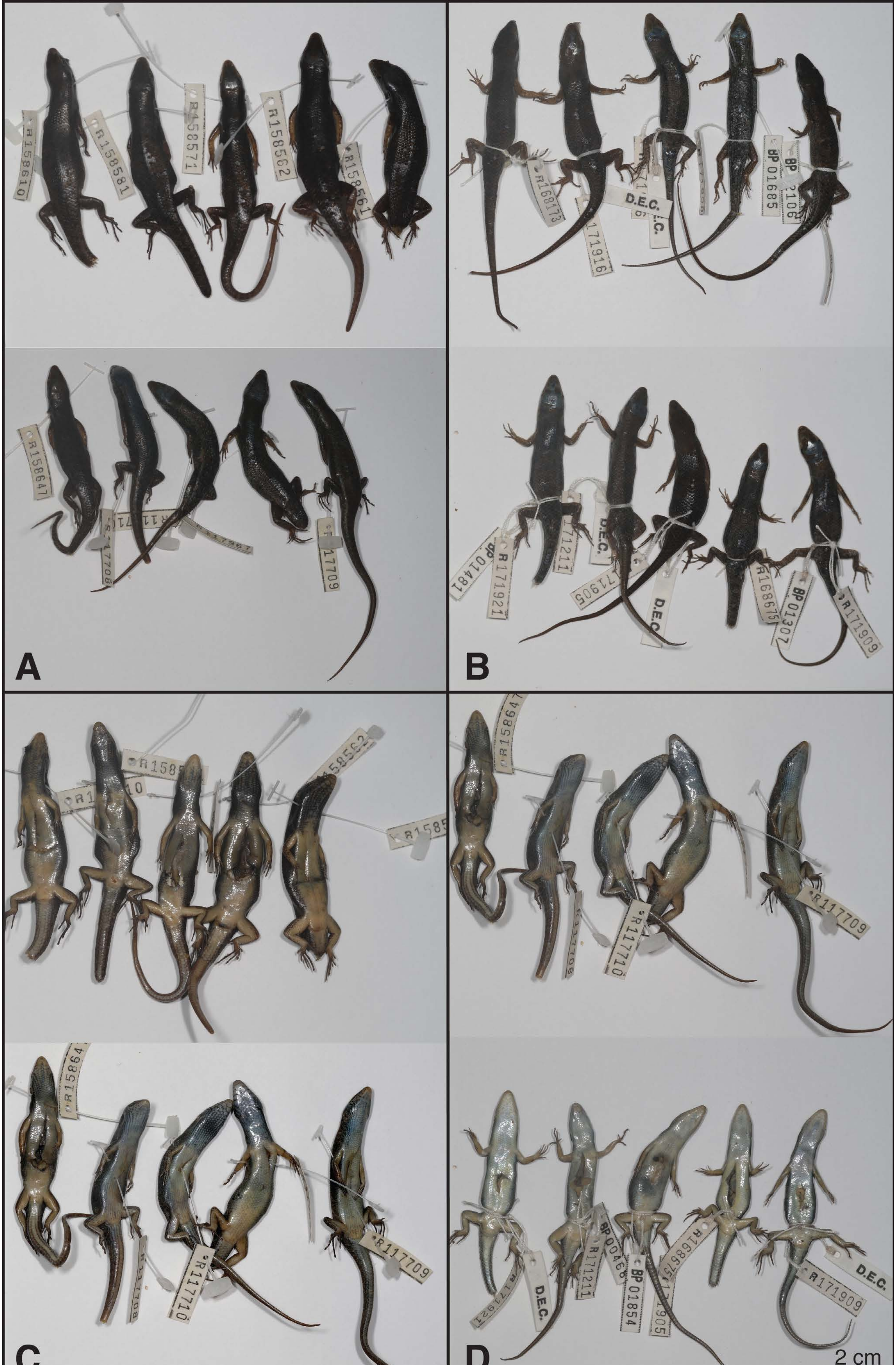

Supplemental Figure S8 – Dorsal and ventral paratype photos for *C. insularis* sp. nov (A, C) and for *C. isostriacantha* sp. nov. (B, D). All photos by Damien Esquerré.
